# Plant Adaptation to Flooding Stress under Changing Climate Conditions: Ongoing Breakthroughs and Future Challenges

**DOI:** 10.3390/plants12223824

**Published:** 2023-11-11

**Authors:** Amna Aslam, Athar Mahmood, Hafeez Ur-Rehman, Cunwu Li, Xuewen Liang, Jinhua Shao, Sally Negm, Mahmoud Moustafa, Muhammad Aamer, Muhammad Umair Hassan

**Affiliations:** 1 Department of Botany, University of Agriculture Faisalabad, Faisalabad 38040, Pakistan; mirzairza65@gmail.com (A.A.); hafeezplansciences.uaf.edu.pk@gmail.com (H.U.-R.); 2 Department of Agronomy, University of Agriculture Faisalabad, Faisalabad 38040, Pakistan; athar.mahmood@uaf.edu.pk; 3 Guangxi Key Laboratory of Water Engineering Materials and Structures, Guangxi Institute of Water Resources Research, Nanning 530023, China; gostli@126.com (C.L.); jinhua20211103@outlook.com (J.S.); 4 Department of Life Sciences, College of Science and Art Mahyel Aseer, King Khalid University, Abha 62529, Saudi Arabia; snsir@kku.edu.sa; 5 Department of Biology, College of Science, King Khalid University, Abha 61421, Saudi Arabia; mfmostfa@kku.edu.sa; 6 Research Center on Ecological Sciences, Jiangxi Agricultural University, Nanchang 330045, China; muhammadaamer@jxau.edu.cn (M.A.); muhassanuaf@gmail.com (M.U.H.)

**Keywords:** aerenchyma cells, flooding, genetic adaptions, hormones, radial oxygen loss

## Abstract

Climate-change-induced variations in temperature and rainfall patterns are a serious threat across the globe. Flooding is the foremost challenge to agricultural productivity, and it is believed to become more intense under a changing climate. Flooding is a serious form of stress that significantly reduces crop yields, and future climatic anomalies are predicted to make the problem even worse in many areas of the world. To cope with the prevailing flooding stress, plants have developed different morphological and anatomical adaptations in their roots, aerenchyma cells, and leaves. Therefore, researchers are paying more attention to identifying developed and adopted molecular-based plant mechanisms with the objective of obtaining flooding-resistant cultivars. In this review, we discuss the various physiological, anatomical, and morphological adaptations (aerenchyma cells, ROL barriers (redial O_2_ loss), and adventitious roots) and the phytohormonal regulation in plants under flooding stress. This review comprises ongoing innovations and strategies to mitigate flooding stress, and it also provides new insights into how this knowledge can be used to improve productivity in the scenario of a rapidly changing climate and increasing flood intensity.

## 1. Introduction

The increasing frequency and intensity of climate change has led to a serious increase in flooding stress [1,2,3]. Temporary and prolonged flooding stress has a significant impact on plant growth and survival, making it imperative for agricultural researchers to identify the involved mechanisms underlying plant adaptation to the aforementioned challenge [4]. Flooding significantly reduces the stomatal conductance, photosynthesis, nitrogen fixation, shoot growth, root development, and nutrient absorption of plants, which results in significant yield losses [5]. Flooding stress also disrupts the gas diffusion between cells and the O_2_ diffusion in plant tissues, which restricts mitochondrial respiration and O_2_ exchange, thereby substantially impairing biochemical and physiological processes in plants [6]. The low oxygen availability under flooding activates the anaerobic microbes, which have a negative impact on plant functioning [7,8] and crop productivity [9]. Understanding current developments and foreseeing the upcoming difficulties in plant adaptation to flooding stress is vital in order to cope with the future challenges of food security and the conservation of agriculture under the changing global climate [10,11,12].

Flooding stress causes oxygen deficiency, which can limit root growth and also leads to root death. Further, water and nutrient absorption and transport are impaired under flooding stress, which causes a reduction in growth and field production [13,14]. Flooding stress also decreases the leaf nitrogen content and impairs the leaf’s water potential, CO_2_ assimilation, and photosynthesis; it also accelerates leaf chlorosis, and senescence may also be observed [13]. Additionally, flooding stress increases reactive oxygen species (ROS) production, which causes oxidative damage and degrades cellular membranes, proteins, and lipids [15]. Water-logging-induced ROS production causes lipid peroxidation, which causes membrane injury, enzyme inactivation, and eventually cell death [16,17].

Plants adopt different physiological, morphological, and biochemical strategies to cope with flooding stress [18,19]. However, the different strategies adopted by plants depend on the alteration of the root structure, the gas exchange mechanism, and metabolic pathways [20,21]. For instance, some plants that are tolerant to low or no oxygen can develop morphological adaptations to compensate for the oxygen deficiency in their roots [22]. Further, plants produce adventitious roots in response to flooding stress, which improves gas transport and nutrient and water uptake and enhances the plant’s survival and productivity [23]. The adventitious roots developed by plants are able to uptake and transport O_2_, making it available to submerged roots [24]. Plants also change their root architecture, which allows them to withstand flooding stress. For instance, in flooded areas, a shallow root system is more beneficial for O_2_ uptake, since the upper layer of soil has more O_2_ compared with lower layers [25]. The maintenance of membrane stability is another important mechanism used by plants to tolerate flooding stress [26]. Plants also activate a vast number of stress response genes, and essential functional proteins are synthesized [27]. For instance, different authors found that plants downregulate photosynthesis-related genes (PsbQ, PsbO, and petF) and light-harvesting chlorophyll protein complex genes (LHCB1, LHCB3, LHCB5, LHCA1, and LHCA4) in response to flooding stress [28,29]. 

The goal of this review is to present a thorough assessment of the current research addressing plants’ ability to adapt to flooding stress in climatic change scenarios. We focus on the causes, effects, and recent developments in genetic, physiological, and ecological aspects of plants (ongoing breakthroughs) in terms of resisting flooding stress. This review will contribute to the development of sustainable methods to reduce the effects of flooding stress on plants in the era of climate change by synthesizing existing knowledge and emphasizing emerging trends.

## 2. Causes of Flooding

### 2.1. Intense Precipitation

**Figure 1:**
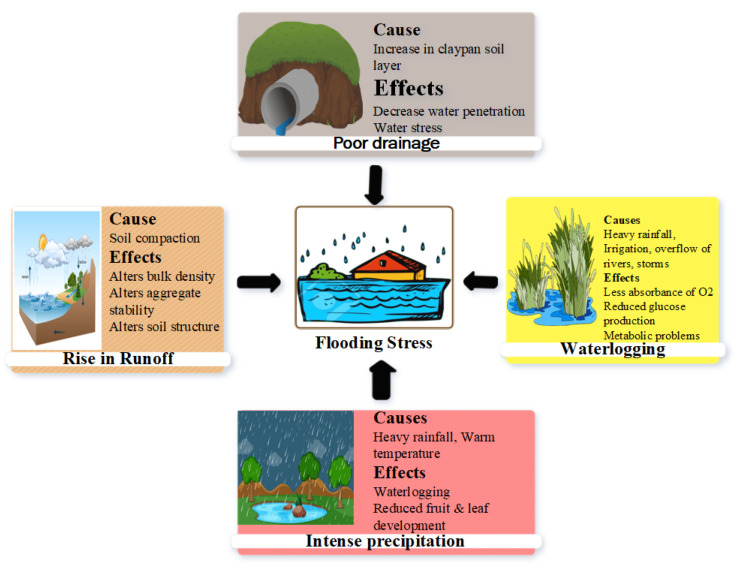
Heavy rainfall, high temperatures, waterlogging, and poor drainage are important causes of flooding stress.

Plants face complex physiological and morphological challenges under flooding stress. High rainfall is the main driver of flooding (Figure 1), and an increasing rainfall intensity worsens the arable land’s productivity [30]. Unexpected rainfall significantly impairs the soil quality, soil saturation, root architecture, nutrient intake, and overall crop yield [19,31]. Westra et al. [32] observed a significant increase in flooding stress on crops due to a high intensity of rainfall. Furthermore, climate change has shifted rainfall patterns and contributed to flooding stress, which hinders crop growth [33]. Extreme weather patterns, alterations in land cover, and a rise in sea levels are the most significant sources of intense precipitation and flooding [34]. The temperature rise enables air to hold more moisture, leading to intense precipitation [32]. Heavy rainfall overwhelms the water absorption ability of soil and causes surface water accumulation [35]. The saturated soil possesses decreased O_2_ availability in the root zone, which leads to flooding stress for crops [36]. Long-term submersion hinders metabolic processes, reduces root respiration and nutrient uptake, and causes asphyxia [31,37]. Additionally, heavy rainfall leads to the loss of fertile topsoil due to soil erosion and thus reduces agricultural productivity [38,39].

### 2.2. Poor Drainage System

A high water table, over-irrigation, rainfall after irrigation, and inadequate drainage increase flooding stress [5]. The inappropriate engineering of the drainage system hinders water removal from the field, whereas a shallow water table also intensifies the waterlogging conditions Figure 1 [40]. An upward-sloping topography in the land also enhances the flooding situation, where an inefficient irrigation system prevails in low-lying soil positions [5,41]. Thus, the installation of an advanced drainage system can substantially reduce the risk of flooding stress in agricultural fields [42,43].

### 2.3. Soil Compaction

Water infiltration decreases with soil compaction caused by heavy machinery (tractor wheel), which also causes stress in field crops. This is due to alterations in soil bulk density and soil particles and disturbances in the soil structure caused by soil compaction [5]. For example, puddled rice cultivation leads to poor drainage conditions, which subsequently leads to flooding for the succeeding (wheat) crop [44]. These factors jointly cause severe flooding stress and impair the plants’ performance. 

### 2.4. Soil Type

The clayey soil is very susceptible to water-logging under heavy rainfall and poor irrigation systems [45,46]. Blessitt [45] reported a high water holding capacity in soil clay particles due to the swelling of the particles, which further impedes the water infiltration in the soil profile, thus creating flooding stress to plants [5]. In addition, the natural soil layers restrict the free drainage of water in the deep soil due to the presence of claypan, which reduces infiltration. The depth of the claypan may range from 10 cm to 40 cm [47,48] and these clay particles may be greater than 460 g/kg [49], leading to a perched water table in the upper soil profile during heavy precipitation in the rainy season [50]. These perched tables lead to short-term flooding stress in the area where water readily infiltrates in sandy soil but accumulates above the compacted clay sub-soil [5].

### 2.5. Snowmelt

Snowmelt also contributes significantly to the development of flooding stress. Snowmelt-induced flooding stress is mainly driven by elevated temperatures. The extent of the relationship between temperature fluctuations, snow accumulation, and melting is critical to the extent of flooding stress [51]. A changing climate exacerbates the snowmelt process and leads to a noteworthy contribution to waterlogging or flooding stress in crops [52]. In this content, agricultural land with a steep slope and no land cover is considered vulnerable to waterlogging and flooding stress [5].

### 2.6. Over-Irrigation 

The excessive application of irrigation can also be an important cause of flooding stress. The excess of water in the root zone causes excessive irrigation, which inhibits gas exchange with the atmosphere and results in an oxygen-deficient environment for the root system [53]. Intensive irrigation exposes the soil to flooding stress, owing to the fact that water seeps through and results in a rise in the soil water table. The intensive method of irrigation exposes soil to waterlogging, and the excessive moisture in the root zone over the field capacity deprives the crops of water and valuable nitrogen. Further, over-irrigation increases the soil salinity, which negatively affects plant growth and development [54].

### 2.7. Ionic Toxicity

Flooding stress also causes a sharp decrease in soil redox potential, which changes the soil’s chemical profile [55]. Flooding stress changes the availability of mineral substances; it causes a reduction in manganese (Mn^4+^), iron (Fe^3+^), and sulfate (SO_4_^2−^) availability and an increase in potentially toxic elements [56]. For instance, Khabaz-Saberi et al. [57] found that flooding stress in wheat increased the concentrations of shoot aluminum (Al), manganese (Mn), and iron (Fe) 2–10 fold as compared to no flooding stress. In addition, Khabaz-Saberi et al. [58] found that flooding stress in wheat increased the concentrations of Al, Mn, and from 3 to 9 fold, thus resulting in a substantial reduction in aboveground biomass and grain yield. Zeng et al. [59] found that flooding stress in barley increased the toxicity of Fe and Mn, which caused a serious reduction in barley growth and yield. 

## 3. Effects of Flooding Stress on Plants 

### 3.1. Seed Germination

Seed germination is the preliminary step towards plant growth and it is controlled by different hormones and abiotic factors [60,61,62,63]. Among these, O_2_ availability has prime importance for seed germination [64,65]. Under an optimal oxygen supply, the reserved starch in the seed is converted into sucrose and produces ATP, which facilitates the germination process [66]. ATP production is significantly reduced during low oxygen availability in flooding situations [4]. Interestingly, the flooding tolerance ability also varies amongst plant species [67]. Rice expresses α-amylase activity under low O_2_ availability that strengthens seeds, allowing them to germinate in flooding conditions [68]. The AMY3 gene is responsible for the α-amylase activity in rice under flooding stress [68]. Further, the calcineurin b-like-interacting protein kinase (CIPK15) aids SnRK1A accumulation, which prompts MYBS1 and AMY3D gene expression to mediate carbohydrate catabolism in flooding stress conditions [68,69]. OsTPP7 is a quantitative trait locus (QTL) responsible for the transcription of the AMY3D gene, and it decomposes starch to provide the ATP necessary for seed germination [4,70]. Although wheat and barley seeds contain high starch content, these cereals are quite sensitive to anoxic (flooding) stress [4,71]. However, this sensitivity can be decreased with the exogenous application of glucose, confirming the role of α-amylase activity towards seed germination in flooding stress conditions [4]. Nevertheless, the molecular-based mechanism responsible for reducing the flooding stress sensitivity still needs to be explored.

### 3.2. Seedling Establishment

The second most important stage in the plant life cycle is seedling establishment, which is greatly influenced by flooding stress [72,73]. The roots are the preliminary organs that face oxygen deficiency and undergo the various phenotypic alterations that seriously impact plant growth and shoot development during flooding stress [74,75]. Flooding significantly reduces the root length and dry biomass of cereals [76,77]. However, rice is associated with the development of adventitious roots (ARs) to obtain O_2_ in *Arabidopsis* [74]. Similarly, *Solanum dulcamara* continues its life cycle in flooding situations due to the presence of ARs [78]. In *Arabidopsis*, the HRE2 gene is responsible for the expression of ARs, which helps to modify the root system through RAP2.12 in anoxic stress conditions [74,79]. RAP2.12 helps to reduce root bending and lowers PIN-FORMED2 accumulation and IAA flux from the root tips in the root zone; this ultimately leads to high IAA in the root tips and slanting growth [79]. 

Agronomically, treatment with calcium peroxide augments seeding establishment by enhancing both the root and shoot length and plant biomass [72]. It provides ATP to germinating seeds by amplifying the α-amylase activity in seeds during flooding conditions [4,72]. Further, the enzymatic (DH, ALDH, and PDC) activity is enhanced in plants facing anoxic respiration [80], whereas exogenously applied calcium peroxide significantly reduces this enzymatic response by enhancing the soluble O_2_ content in water during flooding stress [72]. Although the oxygen availability in plants under flooding stress is enhanced by calcium peroxide application, it is still necessary to identify the adverse effects of flooding on early stand establishment. 

### 3.3. Reproductive Growth 

The crop quality and final yield are largely determined by the successful completion of the plant reproductive stage (Table 1). Under flooding stress, flower and bud initiation and fertilization are affected, which lowers the grain count, size, and weight along with the starch content [81]. Soluble starch synthase is responsible for catalyzing amylopectin, which is downregulated under flooding stress [4]. However, granule-bound starch synthase-I, a key enzyme responsible for amylose synthesis, is upregulated [81]. He et al. [82] found that calcium peroxide treatment enhanced the photosynthetic activity and fruit size of cucumbers during flooding stress conditions. Further, an abundant supply of nitrogen was found to enhance the leaf area, the activity of photosystem II, and the overall maize yield under flooding stress [83].

### 3.4. Crop Yield

Flooding stress is a serious form of abiotic stress and it can cause yield losses (Table 1) of up to 70% [84]. However, yield losses largely depend on the stage of plant growth (Table 1) and the intensity of flooding stress [54,85,86,87,88,89,90]. For instance, Ploschuk et al. [91] reported that flooding stress at the vegetative growth stages in barley and canola caused more yield losses as compared to flooding stress at the reproductive stage. These authors found that flooding stress reduced the spike and seed production, therefore causing a reduction in productivity. Chen et al. [86] also found a significant decrease in crop yield due to flooding stress in the vegetative stage, while Kaur et al. [5] found that flooding stress at the reproductive stage in soybean caused a yield reduction of up to 20–29%. Likewise, Singh et al. [90] reported a significant decrease in maize and soybean yields due to flooding stress at the vegetative stage as a result of fewer spikes and reduced seed production. 

**Table 1:** Responses of different plant species to flooding stress.

Plant Species	Response	Reference
Rice	Restricted shoot elongation as well as carbohydrate consumption.	[92]
Wheat	The ratio of root/shoot significantly declines.	[26]
Maize	Inhibited maize growth, resulting in declines in plant height, ear height, dry weight, leaf area index, and grain yield.	[93]
Soybean	Root growth of soybean is significantly suppressed.	[94]
Tomato	Flooding stress reduces tomato growth.	[95]
*Rumex palustris*	Inhibition of auxin transport, suppressed ethylene-induced AR formation.	[96]
*Triarrhena sacchariflora*	Activity of anti-oxidative enzymes POD and superoxide dismutase (SOD) in roots increases first and then decreases.	[97]
*Arabidopsis*	Starch content in rosette leaves is reduced with the extension of submerged time during the night, and glucose content declines.	[98]

Furthermore, flooding water physically disturbs the crop foliage via water flow and abrasion from suspended particles [20]. Under extreme stress, the plant starts wilting, whereas high soil moisture and a humid environment increase the possibility of disease and infection [99]. Scab in wheat (*Fusarium graminearum*), common smut (*Ustilago maydis*) in corn, and downy mildew (*Sclerophthora macrospora*), gray leaf spot (*Cercospora zeae-maydis*), and sudden death syndrome (*Fusarium virguliforme*) in soybean are the most commonly reported diseases and infections in plants during flooding stress conditions [5].

### 3.5. Soil Properties

An ample amount of oxygen in the atmosphere, which easily enters the soil, is necessary for microbial decomposition [5]. Oxygen is a strong electron acceptor and is readily reduced by NO_3_−, Mn^4+^, Fe^3+^, SO_2_−, and CO_2_ in anaerobic conditions during flooding (ADD REF). Redox potential (Eh) refers to microbial activity; therefore, it is vital to ensure oxygen availability in the soil composition [100]. Measuring the redox potential (ranges) indicates the presence of NO^3−^ in either oxidized or reduced form, which may lead to nitrogen losses (N_2_O emission via denitrification). A typical redox potential value varies between −300 and +900 mV, while the Eh of reduced soil falls within the range of −300 mV to +400 mV [101]. Further, the Eh of aerobic (oxic), sub-aerobic, and anaerobic (anoxic) soils in >414, 414–120, and more than 120 mV, respectively [102]. Denitrification starts at 220–280 Eh mV and causes significant nitrogen loss in the environment [103]. The organic matter percentage, electron acceptors, soil pH, and temperature during flooding affect the soil Eh and are considered to control nitrogen losses [104]. In a study, the frequency of O_2_ reduction increased from anaerobic soil during flooding as the soil Eh decreased by 50 mV each day [105]. Moreover, reduced redox potential was observed with decreased O_2_ from flooded soil due to anaerobic conditions [106].

Kögel-Knabner et al. [100] reported that the soil pH varied with the soil redox potential in flooded soil. The accumulation of more CO_2_ from alkaline flooded soil decreased the soil pH, while the pH increased in acidic coil with high proton consumption [107]. Flooding conditions alter the soil pH depending on the amount of OM, the soil type, and the microbial concentration in the soil [108]. Kögel-Knabner et al. [100] observed a variation in Eh of 59 mV, which disturbed the soil pH by one unit [105]. In contrast, another study found that the was no significant effect of flooding on the soil pH [106]. Moreover, the flooding effect helps to control soil chemical reactions, and the temperature of waterlogged soil was observed to be higher than that of non-stressed soil particles [105,106]. Whereas Zurweller et al. [109] could not confirm the temperature and soil Eh variations in flooding stress, recent studies have reported a significant impact of flooding stress on the soil temperature, pH, Eh, and subsequently nitrogen transformation and its availability to crops due to high N losses [105].

Flooding stress reduced the soil nitrogen availability to plants [106,108]. Unger et al. [106] observed a nitrate reduction from 3 to 5 weeks in waterlogged soil, and Kongchum [108] noticed a continuous decline in nitrate concentration with each day of flooding. They further reported 61% less NO_3_-N from flooded soil compared to normal soil. This significant reduction was due to the large nitrogen losses caused by increased soil runoff, nitrate leaching, and denitrification under flooding stress [5].

Surface runoff and soil erosion must be controlled as they work similarly to enhance nitrogen losses from agricultural fields (ADD REF). More than 5% of applied nitrogen losses are caused by surface runoff (ADD REF). Further, heavy rainfall accounts for 44% of the total nitrogen and 46% of the NO_3_-N losses from agricultural lands, indicating the role of the precipitation amount and its intensity in the nitrogen losses associated with surface runoff [5]. The conversion of NO^3−^ to any gas representing nitrogen (N) or directly into N molecules is known as the denitrification process, and this process is mainly controlled by various autotrophic and heterotrophic bacteria, called denitrifiers, which are activated under the anoxic conditions that develop during waterlogging or flooding stress.

2NO− 3 → 2NO− 2 → 2NO− → N_2_O → N_2_

Clayey soils are highly susceptible to nitrogen losses and therefore more denitrification process takes place in such soils [5]. A perched water table forms in the lower layer of clayey soil with less hydraulic conductivity [50]. Reduced aerobic nitrogen transformation in clayey soil is found due to its poor drainage, and therefore high N_2_O emissions occur in flooding conditions [108]. Kongchum [108] reported high (1.1 to 2.6%) nitrogen losses from flooded soil as compared to normal soil, where nitrogen was lost by 0.03–0.04% [5]. These results correspond with the findings of Allen et al. [110], who stated a positive correlation between waterlogging and water-filled pore spaces and nitrous oxide (N_2_O) emissions from flooded soils [110].

Nitrate leaching entails the downward movement of NO_3_-N and is considered fundamental in the soil environment [111]. Nitrate is highly soluble; therefore, it is highly susceptible to leaching losses [112]. Approximately 10–40% of nitrogen is lost through the leaching process depending on the nitrogenous fertilizer type, application rate, soil characteristics, and temperature and water availability in the soil [113,114]. Advanced studies of nutrient conservation revealed that the microbial population, soil type, and plant root structure can interact to augment nitrogen utilization while lowering its leaching losses [115]. Water percolation and infiltration are significantly lower in clayey soils and represent a positive interaction between soil pores, plant roots, and microbes; thus, less nitrate leaching takes place [116,117]. Moreover, cutting-edge research elucidated the use of emergent technologies including isotopic tracing and molecular biology techniques to understand the flow pathways of leached nitrogen in deep soil horizons [117,118]. Flooding and waterlogging have also received increased attention in the past decade. Modern research has shown high NO_3_^-^N leaching in sandy soils owing to their low CEC [119]. Additionally, ongoing research using the modeling and remote sensing approaches is expected to provide a better understanding of the impact of flooding stress on nitrogen losses [120].

## 4. Adaptations of Plants to Flooding Stress

### 4.1. Morphological and Anatomical Adaptations

**Figure 2:**
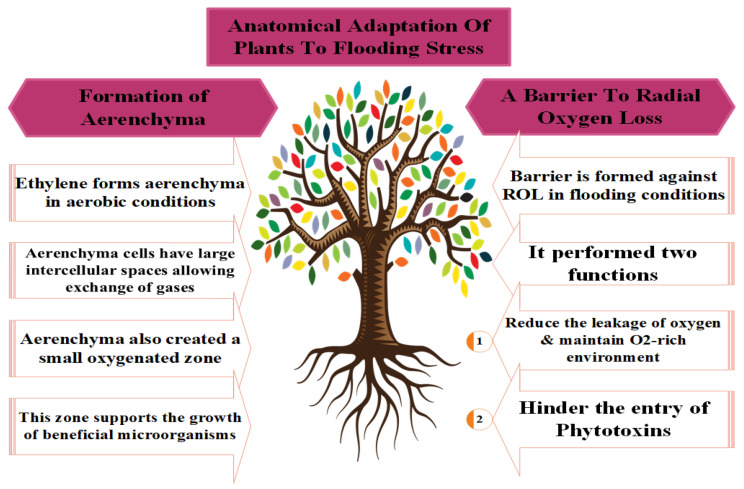
Different adaptations used by plants to counter the toxic effects of flooding stress.

Plants exhibit different morphological and anatomical adaptations to counter the toxic effects of flooding stress (Figure 2). The photosynthesis and respiration rates of crops decrease owing to the low diffusion of O_2_ and CO_2_ in the plant body, except in rice, which develops ARs to adapt to anoxic stress. Conversely, other plants adopt specific morphological modifications to avoid a disrupted energy balance due to the low oxygen diffusion in the root respiratory system under flooding stress [12]. This modification includes AR development, elongation of the apical meristem, air film generation in the upper cuticle, and the creation of a barrier to radial oxygen loss (ROL) [121,122,123,124].

Amongst these, AR development was most commonly observed by [24]. Meanwhile, ref. [125] identified the locations (internodes of hypocotyl or at stem base) of ARs where the respiration and absorption of nutrients occur in plants during anoxic stress conditions [126]. Newly developed ARs have more aerenchyma cells, which increase O_2_ intake and diffusion [96]; thus, ARs successfully replace the primary root system under hypoxia conditions and maintain the plant’s normal growth, development, and metabolism [74].

The oxygen-deficient conditions resulting from flooding stress degrade the cortical cell, leading to programmed cell death and producing tissue cavities and aerenchyma cells responsible for root respiration under stress conditions [127]. Yamauchi et al. [122] also noted the continuous physiological metabolic processes of plants under waterlogged conditions. However, a significant amount of radial oxygen leakage (ROL) takes place in the intracellular spaces during oxygen transfer via aerenchyma cells in root respiration (Figure 2) [122]. Further, plants develop a specialized barrier to control ROL, which diverts the O_2_ molecules in and around the root tips [121]. 

Low oxygen escape syndrome (LOES) is another key adaptation of plants that occurs through an increased apical meristem during flooding stress. This enlargement of the tender stem allows the plants to escape from the anoxic conditions and acquire contact with air for optimum O_2_ intake [128]. Ethylene (ET) production under flooding conditions triggers gibberellic acid (GA) production, which enlarged the intermodal distance in rice [128]. Moreover, the formation of a gas film protects plants from the oxygen-deficient conditions that develop during flooding [129]. Pedersen et al. [130] observed that the net photosynthesis rate of waterlogged rice was only 20% after the removal of an artificial gas film. The gas film maintained the respiration and photosynthetic processes by promoting the O_2_ entry at night and CO_2_ during the day [128].

### 4.2. Photosynthetic Adaptation

Flooding stress causes the closure of stomatal conductance, increased stomatal resistance, and reduced CO_2_ intake, which affects plant photosynthesis [131]. Continuous flooding further reduces enzyme activity, chlorophyll synthesis, and leaf senescence and lowers overall crop productivity [132,133]. Moreover, the loss of pigments (a, b, and carotenoids) and their composition in leaves lowers the photosystem’s efficiency during flooding stress [134]. The expression of the RuBisCo enzyme is mainly controlled by the RuBisCo activase gene and is involved in photosynthesis and photorespiration; it was found to be downregulated in cotton under flooding stress [134]. Sucrose is one of the main products of photosynthesis and its translocation from source to sink was greatly reduced under flooding stress [134]. All these negative changes reduce crop growth and development. Plants undergo a metabolic adaptation involving the secretion of the sucrose synthase enzyme responsible for sucrose breakdown, which is necessary for cellulose biosynthesis to cope with flooding stress [135].

### 4.3. Respiratory Adaptations

Low oxygen availability during flooding stress causes a significant decline in root respiration [136]. The amount of dissolved O_2_ in waterlogged soil was observed to be less than 0.05 mmol/m^3^ compared to the oxygen available (0.23 mol/m^3^) in cultivated soil [137]. Oxygen (O_2_) serves as an electron acceptor in the electron transport chain (ETC), and a decrease in the O_2_ amount reduces ATP generation and leads to a decline in mitochondrial respiration [19,137]. In this context, the glycolysis and ethanol fermentation processes can be alternated as energy sources to ensure continued plant functioning under high water stress [138]. However, the amount of energy produced through the above-mentioned processes is quite low, as only 2 mol of ATP is produced, compared to 36 mol ATP production via the tricarboxylic acid (TCA) cycle [138]. Thus, plants need to amplify the adopted processes during flooding stress to meet the energy demand for normal functioning [12]. 

Pyruvate fermentation is used as an alternate energy source by plants through lactate dehydrogenase (LDH) or pyruvate decarboxylase (PDC), which convert pyruvate into acetaldehyde to reduce ethanol via the alcoholic dehydrogenase (ADH) enzyme [139,140,141]. Borella et al. [142] also noticed the upregulation of ADH and PDC as an alternate source of energy used for respiration in waterlogging-tolerant crops during stress conditions. Further, many researchers have described a similar mechanism in water-logging-tolerant genotypes of cucumber, cotton, and soybean, allowing them to respire under temporary flooding stress [143,144]. Tougou et al. [145] observed the upregulation of the GmADH2 gene in transgenic soybean cultivars during seed germination under flooding stress conditions. Increased flooding stress tolerance was observed in transgenic *Arabidopsis* with the overexpression of the kiwifruit PDC1 gene [146]. All these results support the notion of adaptive mechanisms using PDC- and ADH-based genes during flooding stress [12]. 

PDC’s efficiency to resist flooding stress increased with the production of lactate dehydrogenase (LDH) in *Arabidopsis* [147]. Further, a reduction in or the absence of LDH promoted the opposite phenotype in *Arabidopsis* during flooding [147]. The transcript abundances of the ethanol dehydrogenase genes ADH1-1, ADH1-2, and ADH1-3, and the PDC genes PDC1 and PDC2, were downregulated in *Petunia* plants in which an ET-responsive element-binding factor, PhERF2, was silenced, whereas they were upregulated in PhERF2-overexpressing plants. In contrast, the expression of the LDH gene was upregulated in PhERF2-silenced lines and downregulated in PhERF2-overexpressing lines. This result suggests that the main pathway for NAD^+^ regeneration in PhERF-overexpressing plants is ethanol fermentation, whereas PhERF2-silenced plants might rely on lactic acid fermentation in response to waterlogging stress [148]. However, glycolysis and ethanol fermentation were observed as a temporary source of energy for root respiration during short-term flooding stress [94]. Meanwhile, alcohol, aldehyde, lactic acid, and anaerobic metabolite production caused cell death during long-term flooding stress [12,94].

### 4.4. Activation of Antioxidant Defense Systems and Osmolyte Accumulation 

Flooding stress induces ROS production, which disrupts ionic homeostasis, membranes, DNA, proteins, and lipids [13,149]. However, plants can overcome flooding by activating antioxidant defense systems and increasing the accumulation of potential osmolytes [150]. Plants activate superoxide dismutase (SOD), catalase (CAT), glutathione reductase (GR), and ascorbate peroxidase (APX), which scavenge the flooding-induced ROS production, thus ensuring plants’ survival under flooding stress [151]. Besides this, plants also activate glutathione (GSH), ascorbic acid (AsA), carotenoids, and tocopherols, which protect membranes and the plant photosynthetic apparatus by scavenging ROS production. Moreover, plants accumulate proline, sugars, soluble proteins, and free amino acids to counter the toxic effects of flooding stress [152]. 

### 4.5. Genetic Adaptation to Flood Stress 

Plants activate different genes and proteins to counter the toxic effects of flooding stress [27]. For instance, plants downregulate photosynthesis-related genes and light-harvesting chlorophyll protein complex genes in response to flooding stress [29]. In a study, Borrego-Benjumea et al. [28] noted the participation of different genes involved in metabolic pathways (glucose and nitrogen metabolism) at the root level in barley. These authors also found that the downregulation of these genes was involved in ROS detoxification and nitrogen and amino acid metabolism. In another study, Tong et al. [153] found an increase in respiratory burst oxidase homolog (RBOH) expression, which regulates the accumulation of ROS under flooding stress. These authors also found different quantitative trait loci (QTL) for flooding tolerance features linked with the root biomass, chlorophyll content, and germination index. 

## 5. Role of Phytohormones against Flooding Stress

**Figure 3:**
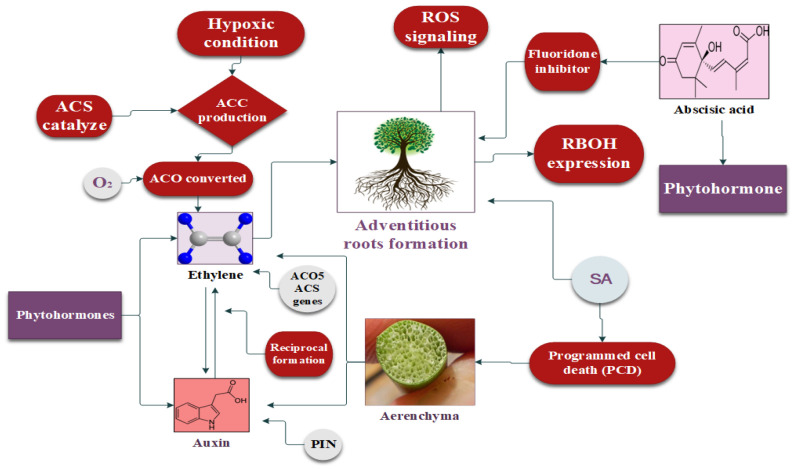
Regulatory mechanisms of phytohormones including ethylene, auxin, and abscisic acid in plant response to flooding stress.

Phytohormones regulate plant functioning to maintain homeostasis and are responsible for normal growth, development, and physiological metabolism under stress (Figure 3) [154,155,156]. The signaling mechanisms created by phytohormones increase the flooding stress tolerance in plants [157,158,159]. The roles of different hormones in inducing flooding tolerance in plants are described below.

### 5.1. Ethylene

Ethylene works as a gaseous hormone in plants, and its diffusion rate is significantly reduced during flooding stress [160]. However, plants have established an adaptive mechanism to accumulate more ethylene to respond to flooding stress. This accumulation is facilitated by the production of 1-aminocyclopropane-1-carboxylic acid (ACC), which serves as the precursor of ethylene production [161]. Under hypoxic conditions, ACC synthase (ACS) catalyzes large quantities of ACC, whereas ACC oxidase (ACO) converts ACC into ethylene in the presence of a small amount of oxygen (O_2_) [162]. Therefore, the continuous transfer of ACC is necessary, from the hypoxic root system to the lower part of the plant’s aerobic region, for a continuous oxidation reaction to produce ethylene [163]. Asgher [164] reported the activation of the ACO5 and ACS genes during waterlogging in *Arabidopsis*, resulting in ethylene biosynthesis taking place. AR development is based on the synthesis of ethylene under flooding stress [163]. In [122], the authors observed the expression of the ACO5 and ACS genes in rice developing ARs and aerenchyma under flooding conditions [165]. Sasidharan and Voesenek [166] reported that ethylene (ET) caused cell death during lysogenic aerenchyma, whereas lysosomal aerenchyma cells developed in wheat, maize, and rice during ET accumulation [122,167]. Moreover, ethylene enhances the biosynthesis of IAA in plants facing flooding stress, which further increases the plant’s vegetative growth, which is responsible for its tolerance of and escape from high-water-stress situations [168]. 

### 5.2. Gibberellin 

Gibberellin (GAs) is essential to control the cell size and number; thus, it plays an important role in the secondary growth and development of plants [169]. Under flooding stress, the GA concentration was found to be increased significantly in waterlogging-tolerant cultivars of soybean compared to stress-sensitive genotypes [170]. Furthermore, the foliar application of GA on peanuts (*Arachis hypogaea*) under flooding stress improved physiological functioning, vegetative and reproductive growth, yields, and root biomass [171]. Similarly, Hong et al. [172] noticed a significant increase in flood resistance due to a decrease in d malondialdehyde (MDA) content in the leaves and roots of rape plants via the exogenous application of GA. Interestingly, GA biosynthesis inhibitors significantly reduced the intermodal elongation in *Oryza sativa* (L.) under flooding conditions [124]. Furthermore, Zhang et al. [173] identified a mutation in the signal transduction gene (OsGID1, OsGID2, OsSPY, OsSEC, OsGAMYB). They also found that GA biosynthesis (Os1, OsCPS2, OsKS2, OsKS5, OsKO2, OsKAO, Os13ox, OsGA20ox1, OsGA20ox2, OsGA20ox3, OsGA3ox1, OsGA3ox2) genes are responsible for lowering the intermodal distance in rice, whereas the foliar application of GA augments stem elongation in rice crops during flooding [12]. The upregulation of GA and its involvement in the SK1/2 gene-mediated pathway enhanced the plant height under flooding stress by stem elongation [124,174]. ET-responsive transcription factor (OsEIL1a) activated the SD1 gene, and the SD1 protein stimulated GA (GA4) synthesis, which further enhanced rice stalk growth [163].

### 5.3. Abscisic Acid 

Abscisic acid (ABA) controls stomatal opening by regulating the size of guard cells in plants [173]. ABA helps to initiate the root aerenchyma cells; therefore, it is recognized as an important phytohormone to respond to flooding stress [175]. Flooding stress decreased the ABA proportion in soybeans by 50% compared to normal conditions [176]. The foliar application of 1 µM ABA decreased the cell development of aerenchyma, suggesting that the development of secondary aerenchyma cells is necessary to reduce he negative regulation of ABA [163]. Moreover, the downregulation of ABA content and adventitious root primordia decreased significantly in *Solanum dulcamara* under flooding stress [177]. Ethylene (ET) built up in the lower portion of the plant stem in waterlogged conditions [56]. As a result, the levels of abscisic acid (ABA) in the adventitious root (AR) primordia as well as the stem primordia decreased significantly [177]. Further, the foliar application of 1 mM ABA reduced AR production during flooding [178]. Conversely, the application of 100 µM of an ABA inhibitor (fluoridone) promoted AR production [179], indicating a negative correlation between ET and ABA in flooding stress conditions. Kim [180] observed a significant decrease in ABA content in soybean after flooding stress, whereas the ABA content was low in waterlogging-resistant cultivars, showing a negative relation between ABA and flooding stress. Meanwhile, significant stem elongation in rice subjected to flooding was observed, with low ABA content, which might have been due to the high ET and GA production under stress [181]. Likewise, ET, along with its precursor (ACC) and inhibitor (1-MCP), was activated simultaneously to control the expression of OsABA8ox1; therefore, ABA was lowered in rice during flooding [182,183]. 

For kiwifruit (*Actinidia deliciosa*), the expression of the AdPDC1 gene was upregulated to encode pyruvate decarboxylase in flooding conditions. This highlights the pivotal role of AdPDC1 in mitigating flooding stress [184,185]. In contrast, a decreased root length and seed germination were noticed due to the overexpression of AdPDC1 in *Arabidopsis* under ABA treatment, showing negative regulation with AdPDC1 in flooding stress [146]. However, ABA accumulates in the aerial parts of the plant during flooding stress and causes stomatal closure under H_2_O_2_ production; thus, water transpiration is significantly reduced under flooding stress [186,187].

### 5.4. Auxin 

Auxin (IAA) is one of the most important growth-promoting phytohormones. The production of ethylene (ET) in waterlogging facilitates the transportation of IAA, and, reciprocally, the accumulated auxin stimulates ethylene (ET) biosynthesis [188]. This reciprocal relationship further promotes the transport of IAA to the flooded regions of the plant, where cell division and AR development take place [189]. In a study, AR growth was inhibited by the exogenous application of an auxin transport inhibitor, 1-naphthylphthalamic acid (NPA), on tobacco, cucumber, and tomato plants under flooding stress [190]. Auxin polar transport carrier protein (PIN-FORMED) is responsible for the transportation of auxin within plants. Treatment with NPA reduced the expression of OsPIN2 and AR development by inactivating the PIN protein in rice [191]. Similarly, mutant *S. dulcamara* lacks PIN expression and, therefore, the transport of auxin was blocked, which caused a significant decrease in the development of adventitious roots (ARs), providing further evidence for the requirement of auxin transport in AR formation [192].

However, certain studies have reported contrasting findings regarding a decrease in IAA levels during stress. For instance, Shimamura [193] observed the development of aerenchyma and ARs after 72 h of waterlogging. Interestingly, physiological tests revealed no noteworthy change in IAA’s endogenous concentration in the hypocotyls compared to controls [194]. This outcome indicates that the presence of accumulated indole-3-acetic acid (IAA) is not a requirement for secondary aerenchyma development in soybean hypocotyls during flooding stress. Moreover, flooding stress can lead to a serious energy deficit by depleting carbohydrates stored in plants. Further, Qi [195] observed a relationship between auxin and sugars, which helped in the commencement and elongation of adventitious roots (ARs) in cucumber during flooding stress. Moreover, photosynthesis supports sugar synthesis in flooding stress by providing energy during the day, which enhances the transportation of auxin and ultimately leads to AR development [196].

### 5.5. Melatonin

Melatonin (MT) is a plant hormone and acts as an antioxidant that enhances plant growth under stress conditions [197,198,199]. MT is well known for its regulatory effects on plants in abiotic stress conditions [196], although few studies have focused on the effect of MT under flooding stress [200]. Fujita and Hasanuzzaman [201] observed an increase in flooding tolerance in plants owing to an increase in the activity of antioxidant enzymes after MT application. Likewise, MT application enhanced the flooding tolerance in apple seedlings [202]. MT enhanced antioxidant enzyme activity, aerobic respiration, and photosystem II efficiency and prevented chlorosis induced by ROS and MDA content in plants under flooding stress [203,204]. The exogenous application of melatonin upregulated the MT biosynthesis genes, such as MbT5H1, MbAANAT3, and MbASMT9 [205]. A significant increase in the previously mentioned genes’ appearance was noticed in MT-treated seedlings, suggesting the crucial role of melatonin towards flooding stress tolerance [171,206]. Flooding stress negatively impacted photosynthetic ability and increased electrolyte leakage and MDA content in *Alfalfa*, whereas MT application (100 µM) enhanced stress tolerance in 6-week-old seedlings [207]. The exogenous application of MT not only improved the physiological and biochemical functioning but also elevated the levels of endogenic MT in stressed plants [208,209].

The decreased expression of ET and signaling genes like ACS, ACO, ERF (Table 2) was noticed in MT-treated seedlings [209]. This suggests a negative correlation between MT and ET production; however, no comprehensive results describe the antagonistic crosstalk concerning MT and ET [163]. Gu et al. [204] reported improved anti-oxidative activity to suppress H_2_O_2_ and lipid peroxidation with 200 µM MT application, which regulated the aerenchyma cells necessary for the respiration of *P. persica* during flooding stress. This was due to the induced mRNA concentration of Ca^2+^ signaling and hypoxia-related ERF VII transcription factor genes [204]. Thus, MT application is suggested to regulate ET homeostasis and flooding stress tolerance [204].

### 5.6. Brassinosteroids

Brassinosteroids (BRs) are naturally produced steroids that improve plants’ resistance to abiotic and biological stresses and promote growth and development [210]. The application of exogenous 24-epi-brassinolide (EBR) facilitated carbohydrate transfer from the leaves to roots in cucumber under hypoxic stress [211]. This process was accompanied by the activation of glycolytic enzymes in the roots, along with antioxidant enzymes, which led to a reduction in ROS production. BR facilitates the loosening and development of the cucumber hypocotyl and promotes the formation of adventitious roots [212]. This process ultimately improved the oxygen availability within the plant body, thereby enhancing its tolerance to hypoxic stress. An exogenous BR treatment activated the Sub1A gene’s (ethylene-response factor (ERF-VII family)) expression, which increased shoot elongation and biosynthetic genes in rice under flooding conditions [213]. In comparison to low oxygen escape syndrome (LOES), the Sub1A rice genotype exhibited BR biosynthesis genes with higher expression, leading to a rise in endogenic BR levels [214]. This elevated BR level stimulated the expression of the GA catabolism gene (GA2ox7), consequently reducing the concentration of gibberellins (GAs) [215]. Simultaneously, GA-mediated responses under submerged conditions were negatively regulated by the GA signal inhibitory factor (SLR1) protein, a member of the DELLA family. As a result, the rice plants’ growth was preserved. Therefore, in the Sub1A rice genotype, BR acted to restrict shoot extension by inhibiting GA biosynthesis and reducing the effect of GA action [216]. 

**Table 2:** Recently discovered genes and relative phytohormones involved in plant responses to flooding stress.

Plant Species	Flooding Type	Gene Name	Function	Reference
Arabidopsis	Waterlogging	LSD1, EDS1, and PAD4	These genes control the formation of *Lysigenous aerenchyma* by regulating the generation of ethylene and ROS.	[217]
Rice	Waterlogging	CIPK15 and SnRK1A	CIPK15 encodes a calcineurin B-like (CBL)-interacting protein kinase that positively regulates the expression of Snf1-related protein kinase 1 (SnRK1A) and functions in rice acclimation to flooding stress by affecting sugar and energy production.	[68]
Maize	Waterlogging	Subtol6	Subtol6 is a major QTL that explains 22% of the phenotypic differences in submergence tolerance within the recombinant inbred lines.	[218]
Wheat	Waterlogging	TaERFVII.1	TaERFVII.1 belongs to the ERF-VII family and functions in the waterlogging tolerance of wheat. The overexpression of TaERFVII.1 increased the survival rate under waterlogging stress	[219]
Barley	Waterlogging	HvERF2.11	The expression of HvERF2.11 can be induced by waterlogging and mediate the waterlogging tolerance of plants through improving some antioxidants’ and ADH enzymes’ activity.	[220]
*Actinidia deliciosa*	Waterlogging	AdPDC1	AdPDC1 encodes a pyruvate decarboxylase that catalyzes the first step in the ethanolic fermentation pathway, and it may function in kiwifruit’s acclimation to waterlogging stress.	[146]
Cucumber	Waterlogging	CsARN6.1	CsARN6.1 encodes an AAA ATPase; transgenic lines of CsARN6.1 showed increased numbers of ARs by enhancing ATPase activity and further affected waterlogging tolerance.	[126]
*Mentha arvensis*	Waterlogging	MaRAP2-4	MaRAP2-4 from *Mentha arvensis* encodes an ERF-I type transcription factor; overexpression of MaRAP2-4 in *Arabidopsis* enhanced its tolerance to waterlogging and oxidative stress.	[221]
Petunia	Waterlogging	PhERF2	PhERF2 regulates the process of programmed cell death and alcoholic fermentation, which enhances waterlogging tolerance.	[148]
*Chrysanthemum morifolium*	Waterlogging	CmSOS1	SOS1 encodes a Na^+^/H^+^ antiporter and may interact with CmRCD1 to mediate plant tolerance to waterlogging stress.	[222]

## 6. Conclusions and Future Prospects

Plants experience a variety of stressful situations during flooding, which depends on the depth and duration of the water. The adaptive characteristics of plants allow them to survive under flooding conditions. These strategies include oxygenating submerged tissues (the positioning of leaves above water to continue carbon fixation; aerenchyma production; and the production of adventitious roots, which act as barriers to prevent radial oxygen loss). To maintain root aeration and prolong water absorption in anaerobic soils, aerenchyma production and the growth of adventitious roots with barriers to radial oxygen loss appear to be the most crucial traits supporting longitudinal oxygen transport. To capture oxygen and continue photosynthesis, a higher proportion of leaves must surpass the water’s surface, which is determined by the length and reorientation of the shoots towards a vertical position. In conditions of low atmospheric evaporative demand, the maintenance of stomata conductance ensures the uptake of CO_2_ for carbon fixation. Meanwhile, in conditions with a high atmospheric evaporative demand, stomata closure can be helpful in controlling plant water homeostasis, which depends on the equilibrium between water losses by transpiration and water uptake by roots. Future research should combine several flooding regimes to examine how plants react to waterlogging and submersion. This would help us to comprehend the advantages and disadvantages of various combinations of characteristics that confer tolerance in circumstances of fluctuating flooding. Therefore, a better understanding of how plants respond to excess water, in the context of increased flooding in the future, would support breeding programs and lead to better management choices for the cultivation of crops and forage species in flood-prone lands.

Flooding stress is becoming more prevalent as a result of continuously changing climatic conditions, which cause significant damage to plants. These challenges will become more serious in the future due to the increased intensity and frequency of severe weather incidents. Some of the key future challenges for plants related to flooding stress are the following. Plants could become submerged for a long period, which will ultimately lead to a shortage of oxygen and nutrients, resulting in substantial yield reductions. Moreover, precipitation patterns will also be substantially disrupted in the coming years due to climate change. As a result, unexpected rainfall events followed by prolonged dry spells could exacerbate flooding stress for plants, making it difficult for them to cope with changing water levels. Flooding events can cause soil erosion and sediment deposition, which can smother plants and disrupt their root systems. The loss of fertile topsoil and the buildup of toxic substances can further challenge plant growth and survival. Flooding stress can severely impact agricultural productivity, leading to reduced crop yields and food shortages. Addressing these challenges will require a combination of efforts, including research on flood-tolerant plant varieties, sustainable land management practices, ecosystem restoration, and policies aimed at mitigating climate change. By adopting these approaches, we can overcome the more prevailing conditions of flooding stress in the changing environmental conditions.

## Data Availability

Not applicable.
